# Military Personals

**Published:** 1918-05

**Authors:** 


### MILITARY PERSONALS

To Army Medical School, Wash., Capt. James C. Davis, Rochester.

To Belleville, Ill., Lieut. Edward F. Meister, Buffalo.

To Camp Gordon, Atlanta, Ga., Major Edward A. Southall, Buffalo.

To Camp Logan, Houston, Texas, Lieut. Ward W. Millias, Rome.

To Camp Shelby, Hattiesburg, Miss., Lieut. Raymond B. Morris, Olean.

To Fort Oglethorpe, Ga., Capts. Maxwell K. Willoughby, Auburn; A. L. Benedict, Buffalo; Lieut. Earl C. Holden, Canastota.

To Fort Riley, Lieut. Andrew J. Goodwin, Ithaca.

To Fairfield, Ohio, Lieut. Milton H. Goldberg, Buffalo.

To Newport News, Va., Lieut. George D. Ubel, Salamanca.

To New York City, Lieut. Walter L. Weeden, Clifton Springs.

To office of Surg.-Gen. Wash., Lieut. John P. Sharp, Niagara Falls.

To Army Medical School for instruction: Lts. Samuel Barone, Raymond Hensel, Porter A. Steele, Buffalo; E. H. Parizot, Cohocton; Roy J. Juhre, Fredonia; John F. Healey, Hornell.

To Camp Hancock, Augusta, Ga., Base Hospital, Lt. Amos J. Minkel, Hamburg; Lieut. Guy Granger, Sherman.

To Camp Lee, Petersburg, Va., Base Hospital, Capt. Willis S. Cobb, Corning; Lt. Antonio L. Barone, Buffalo.

To Camp Upton, L. I., Base Hospital, Capt. Walter H. Vosburg, Dunkirk.

To Curtis Bay, Md., Lt. G. J. Busck, Westfield.

To Ft. Oglethorpe, Ga., for instruction, Capts. Chas. H. Gallagher, Ithaca; Myron B. Palmer, Rochester; Lt. Francis Argus, Rochester; Lt. Samuel Grionstein, Lt. Frederick E. Strozzi, Buffalo.

To Neurologic Institute, N. Y. City for instruction in orthopaedics, Lt. Leonard S. Nolan, Syracuse.

To Rockefeller Institute and on completion to return to Camp Greene, Capt. Coreen T. Graham, Rochester.

To Williamsburg, N. Y. for duty, Lts. Frank Kruse and

Chas. C. Panzarella, Buffalo; Carl G. Sehwan, Danville; Jacob Sacha, Rochester.

Major Francis E. Fronczak, Health Commissioner of Buffalo, spoke on the Pole in America at the meeting of the Civic Education Assn. Apr. 2 shortly before entering upon active military service. Dr. Franklin C. Gram, for many years Registrar of Vital Statistics will act as Health Commissioner during Major Fronczak's absence.

Lt. Col. J. George Adami of Montreal, our Associate Editor writes from London, where he is stationed at present.

To Camp Dodge, Des Moines, Iowa, Lieut. David R. Mellen, Rochester.

To Camp Sevier, Greenville, S. C., Major Frederick J. Barrett, Buffalo.

To Fort Porter, N. Y., Lt. Barton E. Hauenstein, Buffalo.

To Hoboken, N. J., Capt. Frederick Terwilliger, Spencer.

To Cornell University, Capt. Samuel A. Munford, Ithaca.

To Rockefeller Institute, Lieut. Harold J. McDonald, Buffalo.

To Camp Devens, Ayer, Mass., Lt. John A. Wentworth, Clifton Springs.

To Camp Upton, Long Island, Capt. Ralph E. Brodie, Albion; Capt. Walter H. Vosburg, Dunkirk.

To Camp May, N. J., Capt. Samuel R. Hurlburt, Lockport.

To Corpus Christi, Texas, Capt. Alfred W. Armstrong, Canandaigua.

To Fort H. G. Wright, N. Y., Lt. Adelbert C. Abbott, Syracuse.

To Fort Oglethorpe, Ga., Capt. James C. Davis, Rochester; Lieuts. Hiram S. Yeklen, Buffalo; Francis J. McCulla, Frewsburg; John A. Johnson, Olean; Porter A. Steele, Buffalo.

To Fort Riley, Lieut. Carl G. Frost, Buffalo.

To Mineola, L. I., Capt. Edwin S. Ingersoll, Rochester.

To Rochester, Base Hospital, Lt. Hiram Randall, Rochester.

To Rockefeller Institute, Lt. Martin E. Nolan, Tonawanda.

